# Null effect of antidepressants on the astrocytes-mediated proliferation of hippocampal progenitor cells *in vitro*

**DOI:** 10.1186/1744-8069-3-16

**Published:** 2007-06-15

**Authors:** Hyoung-Gon Ko, Sung Joong Lee, Hyeon Son, Bong-Kiun Kaang

**Affiliations:** 1 National Creative Research Initiative Center for Memory and Institute of Molecular Biology and Genetics, Department of Biological Sciences, College of Natural Sciences, Seoul National University, San 56-1 Silim-dong, Gwanak-gu, Seoul 151-747, Korea; 2 Dental Research Institute and Department of Oral Physiology, College of Dentistry, Seoul National University, 28 Yonkon-dong, Chongno-gu, Seoul 110-749, Korea; 3 Department of Biochemistry and Molecular Biology, Hanyang University College of Medicine, 17 Haengdang-dong, Sungdong-gu, Seoul 133-791, Korea

## Abstract

**Background:**

It is well known that antidepressants increase neurogenesis in the dentate gyrus of the hippocampus. The increase of neurogenesis might contribute to the behavioral effects of antidepressants. However, the mechanism by which antidepressants increase hippocampal neurogenesis is largely unknown. It has been recently reported that astroglia induce the neurogenesis of the hippocampal neural progenitor cells (NPCs). Therefore, we hypothesized that antidepressants may act on astrocytes, and this in turn induces neurogenesis of NPCs.

**Results:**

To examine this hypothesis, we used two co-culture systems, i.e., a contact-independent Banker culture and a contact-dependent overlay co-culture. In both of these systems, in comparison with naïve astrocytes, antidepressant-treated astrocytes did not further increase the proliferation of NPCs.

**Conclusion:**

These results suggest that astrocytes increase the proliferation of hippocampal NPCs, however, this may not be directly involved in the antidepressant-induced proliferation of NPCs.

## Background

Depressive disorder is a chronic, life-threatening illness that may be caused by genetic and environmental factors such as stress [1]. Of all the brain regions related to depressive disorders, the hippocampus has received the most attention because many patients suffering from depressive disorders exhibit atrophy in the hippocampus, which regulates the stress response by participating in the hypothalamic-pituitary-adrenal (HPA) axis [2,3]. Recently, it has been reported that antidepressant treatment increases neurogenesis in the dentate gyrus of the hippocampus, and this increased neurogenesis is required for the behavioral effects of antidepressants [4,5]. However, the mechanism by which antidepressants induce neurogenesis in the hippocampus is largely unknown.

It has been well known that astroglia support the functions of neurons. However, a number of recent studies have also shown that astroglia actively control many neuronal functions and development; moreover, this is related to many neurological disorders [6-11]. Many antidepressants are serotonin or norepinephrine reuptake inhibitors [12]. Astrocytes express a serotonin transporter and a norepinephrine transporter [13,14], and this raises the possibility that these cells may be activated and play a role in the action of antidepressants. Moreover, it was recently reported that astroglia induce neurogenesis by increasing the proliferation and differentiation of neural progenitor cells (NPCs) in the dentate gyrus of the hippocampus [15].

We thus hypothesized that when astrocytes are activated by antidepressants, they may release factors that can induce neurogenesis in the dentate gyrus of the hippocampus. In addition, neurogenesis induced by astrocyte-releasing factors may be involved in the behavioral effects of antidepressants. To examine our hypothesis, we used two types of astrocyte-NPC co-culture systems and measured the proliferation of NPCs by using BrdU immunocytochemical analysis.

## Results and Discussion

**Figure 1:**
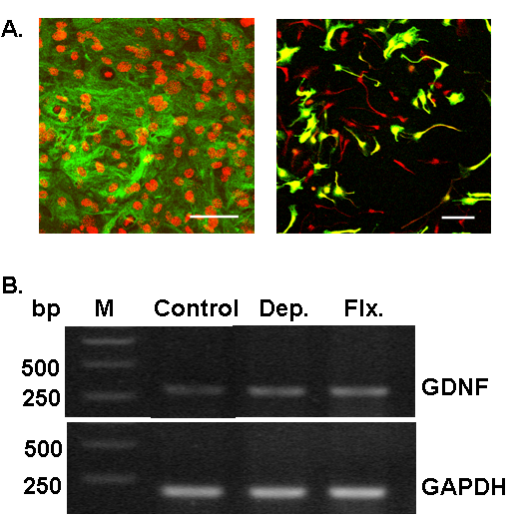
**Cultured hippocampal astrocytes, NPCs and the expression of GDNF mRNA in antidepressant-treated astrocytes**. (A) Cultured hippocampal astrocytes and GFP expressing NPCs were stained with GFAP (left, green) and nestin (right, red) antibody, respectively. Astrocytes were counterstained with PI (left, red). NPCs were stained after the transduction with GFP-expressing retrovirus. Scale bar, 50 μm (left) and 100 μm (right). (B) The results show the RT-PCR analysis of GDNF mRNA in astrocytes. The PCR products of the GDNF and GAPDH mRNAs had sizes of approximately 300 and 180 bp, respectively. GAPDH was used as the internal control. The GDNF mRNA level was increased in astrocytes treated with 10 μM of desipramine or fluoxetine. The experiments were repeated twice to reproduce the similar results. M, size marker. Dep., desipramine. Flx., fluoxetine.

Before we examined our hypothesis, we identified cultured astrocytes and NPCs by immunocytochemistry. As shown in Fig 1A, astrocytes were glial fibrillary acidic protein (GFAP)-positive (astrocyte marker, left panel) and NPCs were nestin-positive (NPC marker, right panel). Antidepressants have been shown to induce the expression of GDNF mRNA [12,16]. To verify the effectiveness of antidepressants in our culture system, we performed RT-PCR for the measurement of GDNF mRNA. Astrocytes were treated with 10 μM of desipramine or fluoxetine. Upon treatment of astrocytes with fluoxetine or desipramine for 48 h, the expression level of GDNF mRNA was higher in cells treated with each antidepressant than that in control cells (Fig. 1B), thereby indicating that these antidepressants were effective at least in inducing GDNF in the astrocytes.

**Figure 2:**
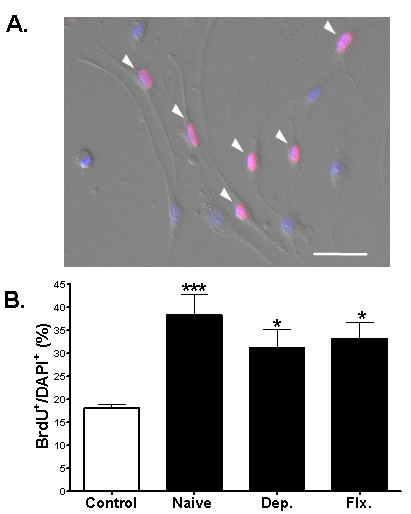
**Effects of antidepressant-activated astrocytes on the proliferation of NPCs in a contact-independent culture system**. (A) Proliferation of NPCs co-cultured with astrocytes using the Banker culture system. The astrocytes were not in contact with NPCs and could affect NPCs only through a diffusible factor. After BrdU immunocytochemical analysis, the nuclei were stained with DAPI (blue). An arrowhead (pink color) marks BrdU-labeled NPCs. Scale bar, 50 μm. (B) Quantification of the proliferation of NPCs. NPCs in control group were not exposed to astrocytes. In comparison with the control group, naïve astrocytes increased the number of BrdU (+) NPCs. However, in comparison with naïve astrocytes, those treated with 10 μM of desipramine or fluoxetine did not further increase the proliferation of NPCs (p > 0.05). Data shown are mean values ± SEM. One-way ANOVA was used for intergroup comparison, Tukey's multiple comparison test was used for post-hoc comparisons.

BrdU is incorporated into the DNA of proliferating NPCs. Thus, the appearance of BrdU immunoreactivity within a cell indicates that the cell was undergoing proliferation at the time upon BrdU was added. Using the BrdU-labeling method, we examined whether antidepressant-treated astrocytes could induce the proliferation of hippocampal NPCs, possibly through diffusible factors. To test this possibility, we used the Banker culture system, which does not allow direct contact between astrocytes and NPCs (Fig. 2A) [17]. The number of BrdU (+) cells was increased in NPCs co-cultured with naïve astrocytes (Fig. 2B; Control: 18.1% ± 0.9%, n = 12; naïve: 38.3% ± 4.6%, n = 10; p < 0.001). This result is consistent with previous findings [15]. However, in comparison with naïve astrocytes, the number of BrdU (+) cells was not further increased in NPCs co-cultured with astrocytes treated with 10 μM of desipramine or fluoxetine (Fig. 2B; Dep.: 31.2% ± 4.0%, n = 10; Flx.: 33.1% ± 3.6%, n = 10; p > 0.05, in comparison with the naïve group). Thus, these results show that the treatment of astrocytes with desipramine or fluoxetine may not affect the proliferation of NPCs via diffusible proliferating factors.

**Figure 3:**
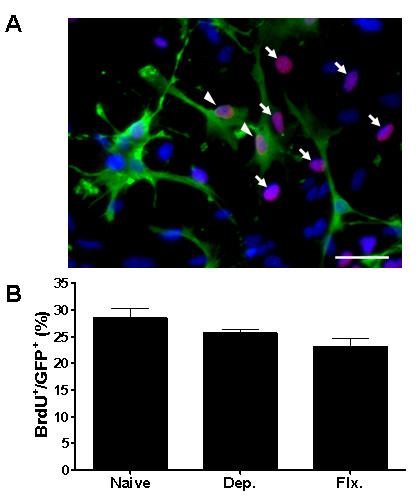
**Effects of antidepressant-treated astrocytes on the proliferation of NPCs in a contact-dependent culture system**. (A) Proliferation of NPCs co-cultured with astrocytes using an overlay co-culture system. After treating astrocytes with antidepressants, NPCs were overlaid on the astrocytes. NPCs were labeled with GFP using a retrovirus. After BrdU immunocytochemical analysis, the nuclei were stained with DAPI (blue). An arrowhead (pink) indicates BrdU and GFP double-positive NPCs, and the arrow (pink) shows BrdU-positive cells. These BrdU-only-positive cells represent astrocytes or NPCs that were not infected with the GFP-expressing retrovirus. Scale bar, 50 μm. (B) Quantification of the proliferation of NPCs. BrdU and GFP double-positive cells were counted. In comparison with naïve astrocytes, those treated with 10 μM of desipramine or fluoxetine did not further increase the proliferation of NPCs. Data shown are mean values ± SEM. One-way ANOVA and Tukey's multiple comparison test were used for data analysis.

On the other hand, the involvement of astrocytes in the proliferation of NPCs could possibly occur in a contact-dependent manner [15]. To test this possibility, we used the overlay co-culture system. In this culture system, astrocytes serve as the substrate cells for NPCs that have been labeled with GFP (Fig. 3A). As shown in Fig. 3B, in comparison with naïve astrocytes, those treated with 10 μM of desipramine or fluoxetine did not increase the number of BrdU and GFP double-positive cells (naïve: 28.5% ± 1.8%, n = 4; Dep.: 25.6% ± 0.7%, n = 5; Flx.: 23.1% ± 1.6%, n = 5, p > 0.05), indicating that desipramine- or fluoxetine-treated astrocytes may not induce proliferation of NPCs in a contact-dependent manner.

In this study, we have examined the possibility that astrocytes are involved in antidepressant-induced proliferation of NPCs. After treated with 10 μM of desipramine or fluoxetine for 48 h, astrocytes were co-cultured with NPCs in a contact-independent or contact-dependent manner. In the former case, although naïve astrocytes induced the proliferation of NPCs in comparison with NPCs that were grown alone, desipramine or fluoxetine treatment of astrocytes did not further increase the proliferation of NPCs (Fig. 2). In addition, treatment with desipramine or fluoxetine did not enhance the proliferation mediated by astrocytes in a contact-dependent manner (Fig. 3). These results, however, cannot exclude the possible involvement of astrocytes in antidepressant-induced neurogenesis. First, the response to antidepressants could be different depending on the mouse strains. For example, C57BL/6 mice are less reactive to stressor and antidepressants than BALB/c mice. These two strains show different 5-HT_2C _pre-mRNA editing responses in the forebrain [18]. Second, we used the experimental scheme to treat BrdU for 24 h because we hypothesized that antidepressants-activated astrocytes might affect the proliferation of NPCs chronically. However, if antidepressants-activated astrocytes affected the proliferation of NPCs transiently, we might be unable to detect the significant effects. Finally, neurogenesis involves three developmental stages: proliferation, differentiation of NPCs, and survival of immature or mature neurons [19]. Although we did not observe the antidepressant-induced proliferating effect of astrocytes, the differentiation of NPCs or survival of immature or mature neurons might be increased by neighboring astrocytes that had been activated by antidepressants. Moreover, a number of signals provided by the microenvironment around the NPCs in the dentate gyrus of the hippocampus have been known to regulate the maintenance, proliferation and differentiation of NPCs [19,20]. Thus, we cannot exclude a possibility that such signals might be additionally required for the astrocyte-induced proliferation of NPCs in the intact brain that had been exposed to antidepressants.

## Conclusion

We hypothesized that astrocytes might be involved in the antidepressant-mediated neurogenesis since it was well known that astrocytes induce the hippocampal neurogenesis and have serotonin or norepinephrine transporter which are targets of the many antidepressants. However, in both contact-dependent and contact-independent co-culture system, astrocytes did not further increase the proliferation of the hippocampal NPCs. Considering that astrocytes are involved in many other neurological functions, more investigations should be carefully performed to elucidate the role of astrocytes on the antidepressant-induced hippocampal neurogenesis.

## Methods

Primary NPCs from the hippocampus of postnatal day 1 or 2 (P1 or P2) C57BL6/J mice were prepared essentially as described previously [21,22]. Hippocampal tissue was dissociated and plated on poly-L-lysine/laminin-coated dishes (25 μg/ml poly-L-lysine; Sigma, 10 μg/ml laminin; Invitrogen) in the presence of Dulbecco's modified Eagle's medium/Nutrient mixture (DMEM/F12) (1:1) containing B27 supplement (without vitamin A), 100 U/ml penicillin, 100 μg/ml streptomycin, and 20 ng/ml bFGF (Gibco BRL). Half of the medium was replaced with fresh medium every 3 days. At least after DIV 6, NPCs were used for co-culturing with astrocytes. For the overlay culture, NPCs were infected with retrovirus to express GFP as a marker at DIV 3.

Primary astrocytes from the hippocampus of P1 or P2 C57BL6/J mice were prepared as described previously [21,22]. Hippocampal tissue was dissociated and plated on a 25 cm^2 ^tissue culture flask (SPL) in DMEM/F12 (1:1), 100 U/ml penicillin, 100 μg/ml streptomycin, and 10% fetal bovine serum (FBS) (Gibco BRL). The medium was replaced every 5 days. When the cells became confluent (DIV 10–11), the flasks were shaken at 200 rpm and 37°C for 3–4 h, and the medium was replaced with fresh medium. Additionally, in case of the overlay co-culture, the cells were treated with cytosine arabinoside (20 μM; Sigma) for 72 h and then washed with phosphate buffered saline (PBS) three times before co-culture.

For the Banker culture, astrocytes were passaged on the poly-L-lysine-coated coverslips (20 μg/ml; Sigma) at a density of 3.7 × 10^4^/cm^2^. On the following day, the cells were treated with 10 μM of fluoxetine (Sigma) or desipramine (Sigma) dissolved in the culture medium for 48 h; this was followed by washing with PBS three times. The concentration of each antidepressant was determined on the basis of the steady-state brain concentrations estimated from a clinical study [23]. With regard to the length of time used for incubation with the antidepressants, we treated the cells with the drugs for the longest possible time, because only chronic antidepressant treatment, not acute, is known to induce neurogenesis. However, the treatment of cells with antidepressants longer than 48 h induced cell death (data not shown) [24]. After washing out the antidepressants, the astrocytes were co-cultured with NPCs that were plated on the poly-L-lysine/laminin-coated glass-bottom dish (10 μg/ml poly-L-lysine, 5 μg/ml laminin) at a density of 1.5 × 10^4^/cm^2 ^in the N2 medium containing 20 ng/ml bFGF.

For the overlay culture, astrocytes were plated on the poly-L-lysine-coated glass- bottom dish at a density of 3.8 × 10^4^/cm^2^. After one day, the cells were treated with 10 μM of fluoxetine or desipramine for 48 h; this was followed by three washes with PBS. After washing out the antidepressants, NPCs expressing GFP were overlaid onto astrocytes in the N2 medium containing 20 ng/ml bFGF.

Twenty-four hours after the co-culture, NPCs were treated with BrdU (10 μM, Banker culture and 2.5 μM, overlay co-culture, respectively) (Sigma) for 24 h. In case of the overlay co-culture, NPCs and astrocytes were treated with BrdU. Finally, co-cultured NPCs were subjected to the immunocytochemical analysis. Briefly, the cells were fixed with 4% paraformaldehyde in PBS. For the detection of BrdU, the cells were treated with 1 N HCl at 37°C for 30 min and then neutralized by washing with 0.1 M sodium borate buffer. After the treatment with HCl, cells were incubated with cold PBT (0.1% Triton X-100 and 0.1% BSA in PBS) for 15 min. After blocking with a preblock solution (2% BSA and 0.08% Triton X-100 in PBS) at room temperature for 2 h, the cells were incubated overnight with the following primary antibodies in a preblock solution: BrdU (1:100, mouse; Sigma), GFP (1:500, rabbit; Abcam), nestin (1:400, mouse; BD biosciences), GFAP (1:400, rabbit; DAKO). After washing with PBT, the cells were incubated with the fluorescence-labeled secondary antibodies for 2 h. Cells were counterstained for 15 min with 10 ng/ml 4', 6'-diamidino-2-phenylindole (DAPI) (Molecular Probes) or 50 μg/ml propidium iodide (PI). Immunofluorescence was visualized using a fluorescence microscope (Carl Zeiss). Fluorescence-positive cells were quantified in 20 fields (×10) per dish either manually (overlay culture) or by using Image J software (Banker culture).

Total RNA was collected using the TRIzol Reagent according to the manufacturer's instructions (Invitrogen). Reverse transcription (RT) reactions were performed using SuperScript™ III Reverse Transcriptase (Invitrogen). cDNA was synthesized from total RNA (2 μg) using a random primer (0.1 μg). One microliter of the RT reaction product was utilized as the template in the PCR with each of the following primer sets. GAT ATT GCA GCG GTT CCT GT and TTC GCA CTG TAG CAG GAA TG were used for GDNF, while TGC ACC ACC AAC TGC TTA and GGA TGC AGG GAT GAT GTT C were used for GAPDH. The number of PCR cycles was set such that it was below saturation conditions and was 30 and 23 for GDNF and GAPDH, respectively.

## Competing interests

The author(s) declare that they have no competing interests.

## Authors' contributions

H-GK carried out all the experiments outlined in the manuscripts, designed the studies and wrote the manuscript. SJL participated in astrocyte culture. HS participated in designing the study and writing the manuscript. B-KK supervised the experiments, participated in the interpretation of data and wrote the manuscript. All authors read and approved the final manuscript.
